# Supplementary material for: Low temperatures lead to higher toxicity of the fungicide folpet to larval stages of Rana temporaria and Bufotes viridis
Source: PLoS One. 2022 Aug 11;17(8):e0258631. doi: 10.1371/journal.pone.0258631 (PMC9371251; doi:10.1371/journal.pone.0258631)
Supplement: S1 Table — (PDF) [file pone.0258631.s001.pdf]

Low Temperatures Lead to Higher Toxicity of the Fungicide Folpet to Larval Stages of *Rana temporaria* and *Bufotes viridis*

Christoph Leeb[1], Laura Schuler[1], Carsten A. Brühl[1], Kathrin Theissinger[1,2]

[1]iES Landau, Institute for Environmental Sciences, University of Koblenz-Landau, Landau, Germany

[2]LOEWE Centre for Translational Biodiversity Genomics, Senckenberg Biodiversity and Climate Research Centre, Frankfurt, Germany

S1 Table. Tested concentrations of Folpan® 500 SC in the acute toxicity tests for two developmental stages of *R. temporaria* and *B. viridis* at different temperatures.

	Developmental stage	T (°C)	Tested concentrations (mg Folpan/L) C_1	C_2	C_3	C_4	C_5	C_6
Rana temporaria	GS20	6	0.00	0.10	0.20	0.25	0.30	0.40
		11	0.00	0.10	0.25	0.35	0.45	0.55
		16	0.00	0.10	0.30	0.45	0.60	0.75
		21	0.00	0.10	0.45	0.55	0.65	0.75
		26	0.00	0.10	0.70	0.90	1.10	1.40
	GS40	6	0.00	0.10	0.50	1.00	1.50	2.00
		16	0.00	0.10	1.00	1.70	2.40	3.10
		26	0.00	0.10	1.60	2.40	3.20	4.00
Bufotes viridis	GS20	6	0.00	0.10	0.40	0.70	1.00	1.30
		11	0.00	0.10	0.40	0.80	1.20	1.60
		16	0.00	0.10	0.60	1.00	1.40	1.80
		21	0.00	0.10	1.20	1.70	2.20	2.70
		26	0.00	0.10	2.30	3.10	4.00	5.00
	GS40	6	0.00	0.10	0.40	0.80	1.20	1.60
		16	0.00	0.10	0.80	1.60	2.40	3.20
		26	0.00	0.10	1.80	2.60	3.40	4.20
