# Supplementary material for: Low temperatures lead to higher toxicity of the fungicide folpet to larval stages of Rana temporaria and Bufotes viridis
Source: PLoS One. 2022 Aug 11;17(8):e0258631. doi: 10.1371/journal.pone.0258631 (PMC9371251; doi:10.1371/journal.pone.0258631)
Supplement: S2 Table — (PDF) [file pone.0258631.s002.pdf]

Low Temperatures Lead to Higher Toxicity of the Fungicide Folpet to Larval Stages of *Rana temporaria* and *Bufotes viridis*

Christoph Leeb[1], Laura Schuler[1], Carsten A. Brühl[1], Kathrin Theissinger[1,2]

[1]iES Landau, Institute for Environmental Sciences, University of Koblenz-Landau, Landau, Germany

[2]LOEWE Centre for Translational Biodiversity Genomics, Senckenberg Biodiversity and Climate Research Centre, Frankfurt, Germany

S2 Table. Number of dead individuals after 48 hours for each replicate in each test.

Species	Devlopement stage	Temperature (°C)	Concentration (mg Folpan/L)	Replicate Nr.	Individuals	Dead after 48 h
B. viridis	GS20	6	0.00	1	5	0
B. viridis	GS20	6	0.00	2	5	0
B. viridis	GS20	6	0.00	3	5	0
B. viridis	GS20	6	0.00	4	5	0
B. viridis	GS20	6	0.00	5	5	0
B. viridis	GS20	6	0.10	1	5	0
B. viridis	GS20	6	0.10	2	5	0
B. viridis	GS20	6	0.10	3	5	0
B. viridis	GS20	6	0.10	4	5	0
B. viridis	GS20	6	0.10	5	5	0
B. viridis	GS20	6	0.40	1	5	0
B. viridis	GS20	6	0.40	2	5	1
B. viridis	GS20	6	0.40	3	5	0
B. viridis	GS20	6	0.40	4	5	0
B. viridis	GS20	6	0.40	5	5	0
B. viridis	GS20	6	0.70	1	5	4
B. viridis	GS20	6	0.70	2	5	5
B. viridis	GS20	6	0.70	3	5	0
B. viridis	GS20	6	0.70	4	5	5
B. viridis	GS20	6	0.70	5	5	3
B. viridis	GS20	6	1.00	1	5	5
B. viridis	GS20	6	1.00	2	5	5
B. viridis	GS20	6	1.00	3	5	5
B. viridis	GS20	6	1.00	4	5	5
B. viridis	GS20	6	1.00	5	5	5

B. viridis	GS20	6	1.30	1	5	5
B. viridis	GS20	6	1.30	2	5	5
B. viridis	GS20	6	1.30	3	5	5
B. viridis	GS20	6	1.30	4	5	5
B. viridis	GS20	6	1.30	5	5	5
B. viridis	GS20	11	0.00	1	5	0
B. viridis	GS20	11	0.00	2	5	0
B. viridis	GS20	11	0.00	3	5	0
B. viridis	GS20	11	0.00	4	5	0
B. viridis	GS20	11	0.00	5	5	0
B. viridis	GS20	11	0.10	1	5	0
B. viridis	GS20	11	0.10	2	5	0
B. viridis	GS20	11	0.10	3	5	0
B. viridis	GS20	11	0.10	4	5	0
B. viridis	GS20	11	0.10	5	5	0
B. viridis	GS20	11	0.40	1	5	0
B. viridis	GS20	11	0.40	2	5	0
B. viridis	GS20	11	0.40	3	5	1
B. viridis	GS20	11	0.40	4	5	0
B. viridis	GS20	11	0.40	5	5	1
B. viridis	GS20	11	0.80	1	5	2
B. viridis	GS20	11	0.80	2	5	5
B. viridis	GS20	11	0.80	3	5	2
B. viridis	GS20	11	0.80	4	5	5
B. viridis	GS20	11	0.80	5	5	4
B. viridis	GS20	11	1.20	1	5	5
B. viridis	GS20	11	1.20	2	5	5
B. viridis	GS20	11	1.20	3	5	5
B. viridis	GS20	11	1.20	4	5	5
B. viridis	GS20	11	1.20	5	5	5
B. viridis	GS20	11	1.60	1	5	5
B. viridis	GS20	11	1.60	2	5	5
B. viridis	GS20	11	1.60	3	5	5
B. viridis	GS20	11	1.60	4	5	5
B. viridis	GS20	11	1.60	5	5	5
B. viridis	GS20	16	0.00	1	5	0
B. viridis	GS20	16	0.00	2	5	0
B. viridis	GS20	16	0.00	3	5	0
B. viridis	GS20	16	0.00	4	5	0
B. viridis	GS20	16	0.00	5	5	0
B. viridis	GS20	16	0.10	1	5	0
B. viridis	GS20	16	0.10	2	5	0
B. viridis	GS20	16	0.10	3	5	0
B. viridis	GS20	16	0.10	4	5	0
B. viridis	GS20	16	0.10	5	5	0

B. viridis	GS20	16	0.60	1	5	0
B. viridis	GS20	16	0.60	2	5	0
B. viridis	GS20	16	0.60	3	5	0
B. viridis	GS20	16	0.60	4	5	0
B. viridis	GS20	16	0.60	5	5	0
B. viridis	GS20	16	1.00	1	5	1
B. viridis	GS20	16	1.00	2	5	2
B. viridis	GS20	16	1.00	3	5	1
B. viridis	GS20	16	1.00	4	5	0
B. viridis	GS20	16	1.00	5	5	2
B. viridis	GS20	16	1.40	1	5	5
B. viridis	GS20	16	1.40	2	5	5
B. viridis	GS20	16	1.40	3	5	2
B. viridis	GS20	16	1.40	4	5	4
B. viridis	GS20	16	1.40	5	5	5
B. viridis	GS20	16	1.80	1	5	5
B. viridis	GS20	16	1.80	2	5	5
B. viridis	GS20	16	1.80	3	5	5
B. viridis	GS20	16	1.80	4	5	5
B. viridis	GS20	16	1.80	5	5	5
B. viridis	GS20	21	0.00	1	5	0
B. viridis	GS20	21	0.00	2	5	0
B. viridis	GS20	21	0.00	3	5	0
B. viridis	GS20	21	0.00	4	5	0
B. viridis	GS20	21	0.00	5	5	0
B. viridis	GS20	21	0.10	1	5	0
B. viridis	GS20	21	0.10	2	5	0
B. viridis	GS20	21	0.10	3	5	0
B. viridis	GS20	21	0.10	4	5	0
B. viridis	GS20	21	0.10	5	5	0
B. viridis	GS20	21	1.20	1	5	1
B. viridis	GS20	21	1.20	2	5	1
B. viridis	GS20	21	1.20	3	5	4
B. viridis	GS20	21	1.20	4	5	3
B. viridis	GS20	21	1.20	5	5	1
B. viridis	GS20	21	1.70	1	5	5
B. viridis	GS20	21	1.70	2	5	5
B. viridis	GS20	21	1.70	3	5	4
B. viridis	GS20	21	1.70	4	5	4
B. viridis	GS20	21	1.70	5	5	5
B. viridis	GS20	21	2.20	1	5	5
B. viridis	GS20	21	2.20	2	5	5
B. viridis	GS20	21	2.20	3	5	5
B. viridis	GS20	21	2.20	4	5	5
B. viridis	GS20	21	2.20	5	5	5

B. viridis	GS20	21	2.70	1	5	5
B. viridis	GS20	21	2.70	2	5	5
B. viridis	GS20	21	2.70	3	5	5
B. viridis	GS20	21	2.70	4	5	5
B. viridis	GS20	21	2.70	5	5	5
B. viridis	GS20	26	0.00	1	5	0
B. viridis	GS20	26	0.00	2	5	0
B. viridis	GS20	26	0.00	3	5	0
B. viridis	GS20	26	0.00	4	5	0
B. viridis	GS20	26	0.00	5	5	0
B. viridis	GS20	26	0.10	1	5	0
B. viridis	GS20	26	0.10	2	5	0
B. viridis	GS20	26	0.10	3	5	0
B. viridis	GS20	26	0.10	4	5	0
B. viridis	GS20	26	0.10	5	5	0
B. viridis	GS20	26	2.30	1	5	0
B. viridis	GS20	26	2.30	2	5	0
B. viridis	GS20	26	2.30	3	5	0
B. viridis	GS20	26	2.30	4	5	0
B. viridis	GS20	26	2.30	5	5	0
B. viridis	GS20	26	3.10	1	5	1
B. viridis	GS20	26	3.10	2	5	0
B. viridis	GS20	26	3.10	3	5	1
B. viridis	GS20	26	3.10	4	5	0
B. viridis	GS20	26	3.10	5	5	1
B. viridis	GS20	26	4.00	1	5	3
B. viridis	GS20	26	4.00	2	5	2
B. viridis	GS20	26	4.00	3	5	1
B. viridis	GS20	26	4.00	4	5	0
B. viridis	GS20	26	4.00	5	5	1
B. viridis	GS20	26	5.00	1	5	5
B. viridis	GS20	26	5.00	2	5	5
B. viridis	GS20	26	5.00	3	5	4
B. viridis	GS20	26	5.00	4	5	4
B. viridis	GS20	26	5.00	5	5	4
R. temporaria	GS20	6	0.00	1	5	0
R. temporaria	GS20	6	0.00	2	5	0
R. temporaria	GS20	6	0.00	3	5	0
R. temporaria	GS20	6	0.00	4	5	0
R. temporaria	GS20	6	0.00	5	5	0
R. temporaria	GS20	6	0.10	1	5	0
R. temporaria	GS20	6	0.10	2	5	0
R. temporaria	GS20	6	0.10	3	5	0
R. temporaria	GS20	6	0.10	4	5	0
R. temporaria	GS20	6	0.10	5	5	0

R. temporaria	GS20	6	0.20	1	5	0
R. temporaria	GS20	6	0.20	2	5	0
R. temporaria	GS20	6	0.20	3	5	0
R. temporaria	GS20	6	0.20	4	5	0
R. temporaria	GS20	6	0.20	5	5	0
R. temporaria	GS20	6	0.25	1	5	0
R. temporaria	GS20	6	0.25	2	5	0
R. temporaria	GS20	6	0.25	3	5	0
R. temporaria	GS20	6	0.25	4	5	2
R. temporaria	GS20	6	0.25	5	5	4
R. temporaria	GS20	6	0.30	1	5	4
R. temporaria	GS20	6	0.30	2	5	2
R. temporaria	GS20	6	0.30	3	5	3
R. temporaria	GS20	6	0.30	4	5	3
R. temporaria	GS20	6	0.30	5	5	1
R. temporaria	GS20	6	0.40	1	5	5
R. temporaria	GS20	6	0.40	2	5	5
R. temporaria	GS20	6	0.40	3	5	5
R. temporaria	GS20	6	0.40	4	5	5
R. temporaria	GS20	6	0.40	5	5	5
R. temporaria	GS20	11	0.00	1	5	0
R. temporaria	GS20	11	0.00	2	5	0
R. temporaria	GS20	11	0.00	3	5	0
R. temporaria	GS20	11	0.00	4	5	0
R. temporaria	GS20	11	0.00	5	5	0
R. temporaria	GS20	11	0.10	1	5	0
R. temporaria	GS20	11	0.10	2	5	0
R. temporaria	GS20	11	0.10	3	5	0
R. temporaria	GS20	11	0.10	4	5	0
R. temporaria	GS20	11	0.10	5	5	0
R. temporaria	GS20	11	0.25	1	5	0
R. temporaria	GS20	11	0.25	2	5	0
R. temporaria	GS20	11	0.25	3	5	0
R. temporaria	GS20	11	0.25	4	5	0
R. temporaria	GS20	11	0.25	5	5	0
R. temporaria	GS20	11	0.35	1	5	0
R. temporaria	GS20	11	0.35	2	5	0
R. temporaria	GS20	11	0.35	3	5	0
R. temporaria	GS20	11	0.35	4	5	1
R. temporaria	GS20	11	0.35	5	5	2
R. temporaria	GS20	11	0.45	1	5	5
R. temporaria	GS20	11	0.45	2	5	5
R. temporaria	GS20	11	0.45	3	5	5
R. temporaria	GS20	11	0.45	4	5	5
R. temporaria	GS20	11	0.45	5	5	5

R. temporaria	GS20	11	0.55	1	5	5
R. temporaria	GS20	11	0.55	2	5	5
R. temporaria	GS20	11	0.55	3	5	5
R. temporaria	GS20	11	0.55	4	5	5
R. temporaria	GS20	11	0.55	5	5	5
R. temporaria	GS20	16	0.00	1	5	0
R. temporaria	GS20	16	0.00	2	5	0
R. temporaria	GS20	16	0.00	3	5	0
R. temporaria	GS20	16	0.00	4	5	0
R. temporaria	GS20	16	0.00	5	5	0
R. temporaria	GS20	16	0.10	1	5	0
R. temporaria	GS20	16	0.10	2	5	0
R. temporaria	GS20	16	0.10	3	5	0
R. temporaria	GS20	16	0.10	4	5	0
R. temporaria	GS20	16	0.10	5	5	0
R. temporaria	GS20	16	0.30	1	5	0
R. temporaria	GS20	16	0.30	2	5	0
R. temporaria	GS20	16	0.30	3	5	0
R. temporaria	GS20	16	0.30	4	5	0
R. temporaria	GS20	16	0.30	5	5	0
R. temporaria	GS20	16	0.45	1	5	1
R. temporaria	GS20	16	0.45	2	5	1
R. temporaria	GS20	16	0.45	3	5	0
R. temporaria	GS20	16	0.45	4	5	0
R. temporaria	GS20	16	0.45	5	5	0
R. temporaria	GS20	16	0.60	1	5	5
R. temporaria	GS20	16	0.60	2	5	5
R. temporaria	GS20	16	0.60	3	5	5
R. temporaria	GS20	16	0.60	4	5	5
R. temporaria	GS20	16	0.60	5	5	5
R. temporaria	GS20	16	0.75	1	5	5
R. temporaria	GS20	16	0.75	2	5	5
R. temporaria	GS20	16	0.75	3	5	5
R. temporaria	GS20	16	0.75	4	5	5
R. temporaria	GS20	16	0.75	5	5	5
R. temporaria	GS20	21	0.00	1	5	0
R. temporaria	GS20	21	0.00	2	5	0
R. temporaria	GS20	21	0.00	3	5	0
R. temporaria	GS20	21	0.00	4	5	0
R. temporaria	GS20	21	0.00	5	5	0
R. temporaria	GS20	21	0.10	1	5	0
R. temporaria	GS20	21	0.10	2	5	0
R. temporaria	GS20	21	0.10	3	5	0
R. temporaria	GS20	21	0.10	4	5	0
R. temporaria	GS20	21	0.10	5	5	0

R. temporaria	GS20	21	0.45	1	5	0
R. temporaria	GS20	21	0.45	2	5	0
R. temporaria	GS20	21	0.45	3	5	0
R. temporaria	GS20	21	0.45	4	5	0
R. temporaria	GS20	21	0.45	5	5	0
R. temporaria	GS20	21	0.55	1	5	0
R. temporaria	GS20	21	0.55	2	5	1
R. temporaria	GS20	21	0.55	3	5	0
R. temporaria	GS20	21	0.55	4	5	0
R. temporaria	GS20	21	0.55	5	5	1
R. temporaria	GS20	21	0.65	1	5	3
R. temporaria	GS20	21	0.65	2	5	1
R. temporaria	GS20	21	0.65	3	5	2
R. temporaria	GS20	21	0.65	4	5	3
R. temporaria	GS20	21	0.65	5	5	0
R. temporaria	GS20	21	0.75	1	5	4
R. temporaria	GS20	21	0.75	2	5	5
R. temporaria	GS20	21	0.75	3	5	3
R. temporaria	GS20	21	0.75	4	5	5
R. temporaria	GS20	21	0.75	5	5	3
R. temporaria	GS20	26	0.00	1	5	0
R. temporaria	GS20	26	0.00	2	5	0
R. temporaria	GS20	26	0.00	3	5	0
R. temporaria	GS20	26	0.00	4	5	0
R. temporaria	GS20	26	0.00	5	5	0
R. temporaria	GS20	26	0.10	1	5	0
R. temporaria	GS20	26	0.10	2	5	0
R. temporaria	GS20	26	0.10	3	5	0
R. temporaria	GS20	26	0.10	4	5	0
R. temporaria	GS20	26	0.10	5	5	0
R. temporaria	GS20	26	0.70	1	5	0
R. temporaria	GS20	26	0.70	2	5	0
R. temporaria	GS20	26	0.70	3	5	0
R. temporaria	GS20	26	0.70	4	5	0
R. temporaria	GS20	26	0.70	5	5	0
R. temporaria	GS20	26	0.90	1	5	0
R. temporaria	GS20	26	0.90	2	5	0
R. temporaria	GS20	26	0.90	3	5	0
R. temporaria	GS20	26	0.90	4	5	0
R. temporaria	GS20	26	0.90	5	5	0
R. temporaria	GS20	26	1.10	1	5	3
R. temporaria	GS20	26	1.10	2	5	2
R. temporaria	GS20	26	1.10	3	5	1
R. temporaria	GS20	26	1.10	4	5	3
R. temporaria	GS20	26	1.10	5	5	1

R. temporaria	GS20	26	1.40	1	5	5
R. temporaria	GS20	26	1.40	2	5	5
R. temporaria	GS20	26	1.40	3	5	5
R. temporaria	GS20	26	1.40	4	5	4
R. temporaria	GS20	26	1.40	5	5	5
B. viridis	GS40	6	0.00	1	3	0
B. viridis	GS40	6	0.00	2	3	0
B. viridis	GS40	6	0.00	3	3	0
B. viridis	GS40	6	0.00	4	3	0
B. viridis	GS40	6	0.00	5	3	0
B. viridis	GS40	6	0.10	1	3	0
B. viridis	GS40	6	0.10	2	3	0
B. viridis	GS40	6	0.10	3	3	0
B. viridis	GS40	6	0.10	4	3	0
B. viridis	GS40	6	0.10	5	3	0
B. viridis	GS40	6	0.40	1	3	0
B. viridis	GS40	6	0.40	2	3	0
B. viridis	GS40	6	0.40	3	3	0
B. viridis	GS40	6	0.40	4	3	0
B. viridis	GS40	6	0.40	5	3	0
B. viridis	GS40	6	0.80	1	3	0
B. viridis	GS40	6	0.80	2	3	1
B. viridis	GS40	6	0.80	3	3	1
B. viridis	GS40	6	0.80	4	3	1
B. viridis	GS40	6	0.80	5	3	0
B. viridis	GS40	6	1.20	1	3	2
B. viridis	GS40	6	1.20	2	3	1
B. viridis	GS40	6	1.20	3	3	1
B. viridis	GS40	6	1.20	4	3	3
B. viridis	GS40	6	1.20	5	3	3
B. viridis	GS40	6	1.60	1	3	3
B. viridis	GS40	6	1.60	2	3	2
B. viridis	GS40	6	1.60	3	3	3
B. viridis	GS40	6	1.60	4	3	3
B. viridis	GS40	6	1.60	5	3	3
B. viridis	GS40	16	0.00	1	3	0
B. viridis	GS40	16	0.00	2	3	0
B. viridis	GS40	16	0.00	3	3	0
B. viridis	GS40	16	0.00	4	3	0
B. viridis	GS40	16	0.00	5	3	0
B. viridis	GS40	16	0.10	1	3	0
B. viridis	GS40	16	0.10	2	3	0
B. viridis	GS40	16	0.10	3	3	0
B. viridis	GS40	16	0.10	4	3	0
B. viridis	GS40	16	0.10	5	3	0

B. viridis	GS40	16	0.80	1	3	0
B. viridis	GS40	16	0.80	2	3	1
B. viridis	GS40	16	0.80	3	3	0
B. viridis	GS40	16	0.80	4	3	0
B. viridis	GS40	16	0.80	5	3	0
B. viridis	GS40	16	1.60	1	3	0
B. viridis	GS40	16	1.60	2	3	0
B. viridis	GS40	16	1.60	3	3	0
B. viridis	GS40	16	1.60	4	3	1
B. viridis	GS40	16	1.60	5	3	2
B. viridis	GS40	16	2.40	1	3	3
B. viridis	GS40	16	2.40	2	3	2
B. viridis	GS40	16	2.40	3	3	1
B. viridis	GS40	16	2.40	4	3	3
B. viridis	GS40	16	2.40	5	3	2
B. viridis	GS40	16	3.20	1	3	3
B. viridis	GS40	16	3.20	2	3	3
B. viridis	GS40	16	3.20	3	3	3
B. viridis	GS40	16	3.20	4	3	3
B. viridis	GS40	16	3.20	5	3	3
B. viridis	GS40	26	0.00	1	3	0
B. viridis	GS40	26	0.00	2	3	0
B. viridis	GS40	26	0.00	3	3	0
B. viridis	GS40	26	0.00	4	3	0
B. viridis	GS40	26	0.00	5	3	0
B. viridis	GS40	26	0.10	1	3	0
B. viridis	GS40	26	0.10	2	3	0
B. viridis	GS40	26	0.10	3	3	0
B. viridis	GS40	26	0.10	4	3	0
B. viridis	GS40	26	0.10	5	3	0
B. viridis	GS40	26	1.80	1	3	0
B. viridis	GS40	26	1.80	2	3	0
B. viridis	GS40	26	1.80	3	3	0
B. viridis	GS40	26	1.80	4	3	0
B. viridis	GS40	26	1.80	5	3	0
B. viridis	GS40	26	2.60	1	3	3
B. viridis	GS40	26	2.60	2	3	3
B. viridis	GS40	26	2.60	3	3	1
B. viridis	GS40	26	2.60	4	3	0
B. viridis	GS40	26	2.60	5	3	1
B. viridis	GS40	26	3.40	1	3	3
B. viridis	GS40	26	3.40	2	3	3
B. viridis	GS40	26	3.40	3	3	1
B. viridis	GS40	26	3.40	4	3	3
B. viridis	GS40	26	3.40	5	3	3

B. viridis	GS40	26	4.20	1	3	3
B. viridis	GS40	26	4.20	2	3	3
B. viridis	GS40	26	4.20	3	3	3
B. viridis	GS40	26	4.20	4	3	3
B. viridis	GS40	26	4.20	5	3	3
R. temporaria	GS40	6	0.00	1	3	0
R. temporaria	GS40	6	0.00	2	3	0
R. temporaria	GS40	6	0.00	3	3	0
R. temporaria	GS40	6	0.00	4	3	0
R. temporaria	GS40	6	0.00	5	3	0
R. temporaria	GS40	6	0.10	1	3	0
R. temporaria	GS40	6	0.10	2	3	0
R. temporaria	GS40	6	0.10	3	3	0
R. temporaria	GS40	6	0.10	4	3	0
R. temporaria	GS40	6	0.10	5	3	0
R. temporaria	GS40	6	0.50	1	3	0
R. temporaria	GS40	6	0.50	2	3	0
R. temporaria	GS40	6	0.50	3	3	0
R. temporaria	GS40	6	0.50	4	3	0
R. temporaria	GS40	6	0.50	5	3	0
R. temporaria	GS40	6	1.00	1	3	0
R. temporaria	GS40	6	1.00	2	3	0
R. temporaria	GS40	6	1.00	3	3	0
R. temporaria	GS40	6	1.00	4	3	0
R. temporaria	GS40	6	1.00	5	3	1
R. temporaria	GS40	6	1.50	1	3	3
R. temporaria	GS40	6	1.50	2	3	3
R. temporaria	GS40	6	1.50	3	3	2
R. temporaria	GS40	6	1.50	4	3	3
R. temporaria	GS40	6	1.50	5	3	3
R. temporaria	GS40	6	2.00	1	3	3
R. temporaria	GS40	6	2.00	2	3	3
R. temporaria	GS40	6	2.00	3	3	3
R. temporaria	GS40	6	2.00	4	3	3
R. temporaria	GS40	6	2.00	5	3	3
R. temporaria	GS40	16	0.00	1	3	0
R. temporaria	GS40	16	0.00	2	3	0
R. temporaria	GS40	16	0.00	3	3	0
R. temporaria	GS40	16	0.00	4	3	0
R. temporaria	GS40	16	0.00	5	3	0
R. temporaria	GS40	16	0.10	1	3	0
R. temporaria	GS40	16	0.10	2	3	0
R. temporaria	GS40	16	0.10	3	3	0
R. temporaria	GS40	16	0.10	4	3	0
R. temporaria	GS40	16	0.10	5	3	0

R. temporaria	GS40	16	1.00	1	3	0
R. temporaria	GS40	16	1.00	2	3	0
R. temporaria	GS40	16	1.00	3	3	0
R. temporaria	GS40	16	1.00	4	3	0
R. temporaria	GS40	16	1.00	5	3	0
R. temporaria	GS40	16	1.70	1	3	1
R. temporaria	GS40	16	1.70	2	3	0
R. temporaria	GS40	16	1.70	3	3	0
R. temporaria	GS40	16	1.70	4	3	0
R. temporaria	GS40	16	1.70	5	3	0
R. temporaria	GS40	16	2.40	1	3	0
R. temporaria	GS40	16	2.40	2	3	3
R. temporaria	GS40	16	2.40	3	3	3
R. temporaria	GS40	16	2.40	4	3	2
R. temporaria	GS40	16	2.40	5	3	0
R. temporaria	GS40	16	3.10	1	3	3
R. temporaria	GS40	16	3.10	2	3	3
R. temporaria	GS40	16	3.10	3	3	3
R. temporaria	GS40	16	3.10	4	3	3
R. temporaria	GS40	16	3.10	5	3	3
R. temporaria	GS40	26	0.00	1	3	0
R. temporaria	GS40	26	0.00	2	3	0
R. temporaria	GS40	26	0.00	3	3	0
R. temporaria	GS40	26	0.00	4	3	0
R. temporaria	GS40	26	0.00	5	3	0
R. temporaria	GS40	26	0.10	1	3	0
R. temporaria	GS40	26	0.10	2	3	0
R. temporaria	GS40	26	0.10	3	3	0
R. temporaria	GS40	26	0.10	4	3	0
R. temporaria	GS40	26	0.10	5	3	0
R. temporaria	GS40	26	1.60	1	3	0
R. temporaria	GS40	26	1.60	2	3	0
R. temporaria	GS40	26	1.60	3	3	0
R. temporaria	GS40	26	1.60	4	3	0
R. temporaria	GS40	26	1.60	5	3	0
R. temporaria	GS40	26	2.40	1	3	0
R. temporaria	GS40	26	2.40	2	3	1
R. temporaria	GS40	26	2.40	3	3	1
R. temporaria	GS40	26	2.40	4	3	0
R. temporaria	GS40	26	2.40	5	3	0
R. temporaria	GS40	26	3.20	1	3	3
R. temporaria	GS40	26	3.20	2	3	2
R. temporaria	GS40	26	3.20	3	3	3
R. temporaria	GS40	26	3.20	4	3	2
R. temporaria	GS40	26	3.20	5	3	2

R. temporaria	GS40	26	4.00	1	3	3
R. temporaria	GS40	26	4.00	2	3	3
R. temporaria	GS40	26	4.00	3	3	3
R. temporaria	GS40	26	4.00	4	3	3
R. temporaria	GS40	26	4.00	5	3	3
